# Sorting out and navigating uncertainty in precision medicine

**DOI:** 10.1007/s40656-026-00726-5

**Published:** 2026-03-24

**Authors:** Cornelius Borck, Simon Lohse

**Affiliations:** 1 Institute for History of Medicine and Science Studies, University of Lübeck, Lübeck, Germany; 2 Cluster of Excellence Precision Medicine in Chronic Inflammation (EXC 2167), Lübeck/ Kiel, Germany; 3 Institute for Science in Society, Radboud University Nijmegen, Nijmegen, The Netherlands; 4 Faculty of Humanities, University of Johannesburg, Johannesburg, South Africa

**Keywords:** Uncertainty paradox, Personalized medicine, Evidence-based medicine, Ontology and epistemology of precision medicine, Interdisciplinary collaboration, Data integration and clinical decision making

## Abstract

Uncertainty has been highlighted as a paradoxical consequence of precision medicine (PM). In the life sciences and medicine, uncertainty is often conceptualized as something that can be clearly delimited and overcome by more information and better understanding derived from more research, more data, better tools etc. Building on the observation of uncertainty in PM and exploring it further by differentiating between socio-technical, epistemological and ontological uncertainty, we claim that at least some forms will likely turn out to be non-transient: Uncertainty in PM does not only result from missing or incomplete information, but also from insights into biological complexity and the destabilization of evidence frameworks. We conclude by discussing some implications for a successful implementation of PM in clinical practice by adequately navigating uncertainty.

## Precision medicine: its promises and the trajectory of its implementation

Precision medicine (PM) is the name for the project to replace medical regimes standardized according to disease classification by individualized therapies taking “into account individual differences in people’s genes, environments, and lifestyles” (The White House, 2015). Initially, PM was widely regarded as a novel, ground-breaking approach to medicine with a multitude of visions for the transformation of healthcare (Collins & Varmus, 2015; Flores et al., 2013; Hood et al., 2012). In addition to new molecular technologies, especially the exponential growth of available data was celebrated, creating new methods for their analysis—for example the opportunity to build “SuperModels,” i.e. patient avatars for pretesting available therapeutic options (Brown, 2015), or to “make precise personalized patient care a clinical reality” (Hodson, 2016), by matching “the right trial to the right patient” (Bahcall, 2015). PM was expected to usher in a new era of medical practice that would not only leave behind the “cookbook medicine” of evidence-based medicine (EBM) but deliver therapeutic interventions precisely tailored to specific patient subgroups or even individual patients (Ashley, 2016; Sankar & Parker, 2017).

As is often the case with visionary approaches, a more sobering wave of debate followed the early enthusiasm. Several authors questioned the grand visions of PM (Lemoine, 2017; Prasad, 2016; Tabery, 2023), raising doubts regarding the adequacy of validation procedures for novel diagnostic and therapeutic approaches (Hey et al., 2020) and concerns as to whether the promises of PM were more than just hype and good PR (Maughan, 2017). In addition, critics warned of overdiagnosis and medicalization of everyday life (Vogt et al., 2019), thereby questioning that tailoring treatments to individual preferences and lifestyle would humanize biomedicine (Tinland, 2022; Vogt & Green, 2020).

A particularly puzzling challenge for PM was highlighted by scholars in philosophy of medicine and the medical humanities who argued that PM would, in fact, introduce more *uncertainty* into medicine (Green & Vogt, 2016; Plutynski, 2022; Tabery, 2023). This uncertainty in PM appears to stem from factors such as the complexity of genomic and epigenetic data, unclear therapeutic implications of ambiguous biomarkers and methodological challenges in interpreting test results from new omics technologies, among other things (Boniolo, 2022; Kerr et al., 2019; Vogt et al., 2019). In light of these issues, there was even talk of a *paradox* of PM (Kimmelman & Tannock, 2018; cf. Hoeyer, 2023), as Henrik Vogt explains:

While seeking to increase precision and certainty in medicine, it [PM] might also create more imprecision and uncertainty. (Vogt, 2022, p. 62)

This observation provides the starting point for this article which will proceed from the assumption that PM indeed generates more uncertainty (and less precision than claimed by its proponents). Building on this insight and in order to advance the philosophical analysis of uncertainty and its extent in PM*,* we suggest *to differentiate and explore various sources of uncertainty*. This is intended as a heuristic for a more precise characterization of uncertainty in PM and will enable us to better understand its different and partially novel sources. What will become apparent through this exploration is that not all types of uncertainty have the same level of recalcitrance: While some forms of uncertainty are transient and will be eliminated thanks to further experience with and progress of PM, others, we suggest, may stand out as *intransient*. This perspective has deep implications for how we evaluate successful research in PM, as this may deconstruct its own vision of precision as individualized certainty into the mere individuality of endless numbers and patterns.

Uncertainty is obviously not a new problem in medicine, but a general feature of clinical practice, if not its core predicament, since at least the beginning of discursive medicine. William Osler famously declared medicine to be “a science of uncertainty and an art of probability” (Osler, 1950, p. 125). The fundamental question for our discussion, i.e. the philosophical issue we want to address here, is to what extent uncertainty merely results from missing or insufficient information or whether medical science itself reveals the matter to be too complex to be truly understood or resolved. The rest of this section hence traces briefly the evolution of uncertainty in the context of the developments from evidence-based medicine to PM. In Sect. 2, we explore uncertainty’s multiple sources in socio-technical, epistemological and ontological perspective. This exploration suggests that in all three domains there are zones of fundamental recalcitrance with regard to reducing uncertainty. Based on this interim result and practical illustrations, we argue that uncertainty in PM has deeper implications for an adequate conceptualization of PM than has generally been acknowledged (Sect. 3): Some new forms of persistent uncertainty will arise as a direct consequence of the type of research conducted in PM and its powerful productivity. We conclude (Sect. 4) by briefly discussing some implications for clinical decision-making and starting points for navigating uncertainty in personalized translational research.

In light of our discussion here, evidence-based medicine can be described as a movement emphasizing the body’s biomedical complexity as intrinsic source of uncertainty and propagating the critical evaluation of available information in search for robust efficacy (Borck, 2021; EBM-working group, 1992; Guyatt et al., 2015; Howick, 2011). EBM recommended “epistemic humility” in form of reliable, statistical measures for the empirical success of specific treatments rather than perfect pathophysiological explanations (McCoy, 2020) in order to legitimize treatment decisions by the probability of success for given diseases as defined by their classification and based on epidemiological data (broadly construed). PM, by contrast, was envisioned to open up the very category of disease and to base decision-making on information specific to the case. In epistemological terms, PM strives to replace relative efficacy by tailored treatments derived from the analysis of large-scale data sets from populations as well as single patients and ideally tested in individualized models (Marchiano et al., 2021). Accordingly, PM was heralded as the vision for overcoming “aleatoric uncertainty” (i.e. uncertainty whether a specific treatment will be successful in the individual case, cf. Scott et al., 2023) caused by large and unwieldy disease entities, by means of genetics, epigenetics and multi-omics.

PM sought to transform uncertainty into epistemic certainty “grounded on mechanistic explanations of molecular interactions, metabolic pathways and biomarkers” (Nardini et al., 2012). In the early vision of PM, uncertainty appeared to be significantly reducible, at least in principle, by introducing a new scientific understanding of the individual (Boniolo, 2017) and by rigorous streamlining of interdisciplinary cooperation between basic science, translational research and clinical decision making (Beckmann & Lew, 2016). This seemed possible by the “momentous theoretical and technological advancements in the biomedical sciences” which had enabled “a deeper understanding” of bodily processes, allegedly accompanied by a “more accurate and effective manipulation of biological systems” (Boniolo & Nathan, 2017, p. 1). Comprehensive cancer care by means of a molecular tumor board provides a good example how this has meanwhile been implemented, turning PM into a reality in oncology at many places (Wahida et al., 2023). We will return to precision oncology in Sect. 4, as it also exemplifies the interplay between the sources of uncertainty we identify. It should be emphasized, however, that cancer is accompanied (if not caused) by genetic mutations, offering thereby a valuable handle for identifying “actionable” targets for intervention on the molecular level (Chin-Yee & Plutynski, 2023; Tsimberidou et al., 2020). Whether and how success in oncology can also pave the way for introducing PM in the treatment of other disease is a different question which we can only start to address in our conclusion.^1^

## Sources of uncertainty in precision medicine

Drawing on the landscape of uncertainty in medicine (Djulbegovic et al., 2011; Han & Djulbegovic, 2019; Scott et al., 2023) and earlier work on forms of uncertainty in PM (Bourret & Cambrosio, 2019; Dam et al., 2022; Plutynski, 2022), we want to explore and characterize uncertainty in PM in more detail and explain it as multifaceted. Following the systematization of types of uncertainty in PM in Lohse (2023), we differentiate uncertainty in PM according to different sources, namely socio-technical, epistemological and ontological—thereby using a standard triad from philosophy of science: The ontological perspective points to assumptions about cells, organisms, diseases, environments, and their particles and interactions; the epistemological perspective focuses on the different scientific approaches for determining and formatting possible findings, for conceptualizing and theorizing relevant information, for deciding evidential questions; and the socio-technical perspective highlights all kinds of factors contributing to success or failure of PM “in real life,” i.e. the material world including availability of appropriate tools, technologies and skills, and the fallibility of interactions in large socio-technical assemblages. Starting with such mundane matters, i.e. socio-technical issues, we will characterize and analyze these three different sources of uncertainty in more detail below. This will prepare the ground for drawing out broader implications for understanding the emergence and persistence of uncertainty in PM.

### Socio-technical uncertainty

A first and obvious, but frequently not fully acknowledged source of uncertainty results from the specific material and technical practices embedded in PM communities. Among PM’s proponents, the real-world problems of implementing PM are typically discussed as a question of proper standards of data collection, issues of strict regimes of data curation and the implementation of transparent protocols for analysis—all of these are already relevant for high-quality research in today’s biomedicine (Colijn et al., 2017; Gansel et al., 2019; Hopp et al., 2018). This is undoubtably true in general and covers important aspects as well as relevant precautionary regulations in data-centric science, but the question arises whether PM can legitimately be envisioned here as seamless “extension” of biomedical research (Beckmann & Lew, 2016). This assumption relates to complex and crucial issues, as PM operates by large interdisciplinary collaborations and (challenging) knowledge integration practices across heterogeneous disciplines, whereas EBM operates mainly within a robust logic of homogenization and standardization across large clinical cohorts, requiring very different precautionary measures. Careful empirical observation and epistemological analysis has pointed to multiple and persistent uncertainties, biases and tensions that get obfuscated and black boxed by the swift implementation of allegedly comprehensive protocols (Leonelli, 2018): Precisely because data-intensive biomedicine interconnects the results from multiple direct, material interactions in routine diagnostics to large data sets for further digital data processing and analysis, all the (error-prone) steps going into the harvesting, filtering and curating of data become absolutely central for the usability and epistemic standing of the data. At the same time, they become opaque, as the data ultimately travel freely once they have been entered into the database or biobank (Hoeyer, 2023; Metzler et al., 2023; Tempini & Leonelli, 2021). As Sabina Leonelli comments, the “process of aligning the informational with the material is specific to big data assemblages and analysis and constitutes one of the foremost scientific challenges of the twenty-first century” (Leonelli, 2018, p. 332). Data integration is well known as technical and practical problem of PM and we do not claim this to be a reason for failure, but it is important to mention that the research in science studies quoted here has revealed how exactly precautionary measures and data protocols may conceal (unresolved) sources of uncertainty, thereby fostering their recalcitrance.

A related socio-technical issue arises from the sheer amount of data. PM generates gigantic masses of data which create typical problems for big data approaches, including manageability, issues with regard to data quality, data noise and the risk of “drowning in data” (cf. Vogt, 2022). Availability of data is the very source of PM’s potential, but its data-driven approach also creates specific problems at the interface of data infrastructures and their use and curation by different and heterogeneous communities. These include issues of data formats and classifications that may be divergent in non-trivial ways as well as standardization practices, including the inconsistent use of metadata (O’Malley & Soyer, 2012). In addition, there is the challenge of how to weigh data that point in different directions with regard to the diagnosis of a specific disease. These challenges can present PM practitioners with significant uncertainties already at the stage of data collection and processing: Which standards should be used as a guide? To what extent can later integration requirements be taken into account in the early stages of data processing (especially when it is not yet clear what other types of new data will need to be integrated)? The pace of the emergence of new diagnostic measures, biomarkers and analytical tools does not only open ever new opportunities for analysis and prediction, but also increases requirements for interoperability (Prosperi et al., 2018).

Next to these challenges, Leonelli (2018) discusses systematic, source-specific differences in the assessment of evidence quality and data reliability, for instance in terms of the contested reliability of micro-array results in different fields of research. All of these issues make meaningful interdisciplinary data integration extremely challenging and may lead to unclear or unreliable results. It is important to note that this is not a point about “mere practicalities” that is easy to overcome as “better” purification or filtering in the different subsystems may increase rather than decrease integration problems, thereby cancelling out potentially significant findings (König et al., 2017). Such difficulties on the micro level are mirrored on the macro where different data practices and standards reflect different research traditions and bio-ontologies associated with different subfields (and they may even be engrained in the specific organization and rationale of national healthcare systems, see Green et al., 2019). This constellation creates uncertainty at a meta level, as it is unclear how best to resolve it. Should core disciplines of PM (which ones?) take on a pioneering role? Must genuinely interdisciplinary data integration practices first be established in different PM sub-fields? Or should bioinformatics play a decisive role, overriding considerations that are anchored in the research traditions of involved life sciences?

In sum, socio-technical uncertainty results from the interoperability required for PM as a big data driven medical practice (Gansel et al., 2019), and the risk is that pursuing PM will ignore or even obfuscate persisting incongruencies (of unknown significance). This would obviously lead to less significant or unreliable results and thereby threatens to make this research futile. It is the trickiest and at the same time most “actionable” source of uncertainty in PM as it is in principle transient, though certainty may turn out to be in-achievable for practical reasons.

### Epistemological uncertainty

Uncertainty in PM has numerous epistemological sources related to conceptual and evidential questions about the type of medical knowledge sought. A first source lies in the drive for greater individualization itself. As PM aims to develop tailor-made therapies for stratified patient groups, the sample size for testing new therapeutic interventions inevitably gets smaller and smaller, reaching its extreme with n = 1 trails, the vision of a truly “individualized” treatment, but with questionable epistemic status (Schork, 2015). This increases the risk of statistical errors and may result in low‐grade evidence regarding the effectiveness of novel therapeutics (Djulbegovic & Ioannidis, 2019; Kimmelman & Tannock, 2018). Moreover, it seems unclear what insights can be drawn from stratified studies with very small sample sizes for other patient groups, given that traditional EBM-style statistical extrapolation is no longer applicable (Vogt, 2022). In short, epistemological uncertainty arises since it is unclear whether PM is in a position to replace the medical epistemology of EBM with a viable alternative (Vogt & Hofmann, 2021).

A related epistemological source of uncertainty runs equally deep, if not deeper. It stems from conceptual questions and a lack of consensus regarding evidence thresholds in PM. Questions revolve around ambiguity in classifying omics findings as significant (Cambrosio et al., 2021; Even Chorev, 2020; Lyon & Wang, 2012)—and around what it means for a finding to be not only biologically significant, but also actionable in terms of the development and application of (new) medical therapies (Chin-Yee & Plutynski, 2023; cf. Nelson et al., 2013). There is a risk here that too many *potentially* relevant biomarkers are identified that may not open up therapeutic options, resulting in more “patients-in-waiting” (Boniolo, 2022). Reservations about the adequacy of existing evidence tools for clinical practice, including genetic risk scores, underscore these epistemological uncertainties (Davis et al., 2018; Green & Vogt, 2016; Horesh Bergquist & Lobelo, 2018). It is worth noting that the persistent evolution of technologies and diagnostic tools in translational PM seems to intensify the described challenges, as this evolution does not only lead to an exponential growth of data, but also to rapidly outdated evidence and, above all, to dynamically changing evidence *standards* (Bourret & Cambrosio, 2019; Newson et al., 2016).

A further epistemological source of uncertainty we wish to highlight relates to challenges of evidence integration in PM. As PM aims to synthesize knowledge about patients on different levels, it needs to integrate evidence of different types, including clinical evidence, omics data and experimental data from pre-clinical research, among other things (such as evidence generated by in silico modeling and wearable health devices). This is not only challenging on a practical level—see the previous section—it also raises significant conceptual uncertainties (Friedman, 2025). For instance: How do we best integrate evidence of diverse types and heterogeneous nature, stemming from very different sources? What kind of knowledge framework do we need for this (Lohse, 2023)? These and related questions do not suggest any simple and, above all, *undisputed* answers that will resolve all ambiguities.

According to the vision of PM as “science of the individual” (Boniolo, 2017), the integration of different types of evidence surfaced merely as a question of precision, appropriate standards and validation procedures; the implicit understanding being that all data relating to a single case should ultimately align if every branch of scientific investigation worked sufficiently accurate. Because of the highly sophisticated nature of techno-scientific research today, however, this position must be dismissed as naïve, as has been shown by work in the philosophy of science in practice: While data from clinical observation and medical case management have to follow clinical guidelines and obey the indicators for appropriate financial reimbursement (Metzler et al., 2023), the modeling of the case in vitro and in vivo has to follow the routines of the respective experimental systems (Rheinberger, 2023). Knowledge is further formatted by the different affordances of animal, cellular, or in silico models which mirror current assumptions (derived from the respective disciplinary expertise) regarding experimental opportunities and assumptions about the significance of specific aspects of diseases, human target systems etc. (Dietrich et al., 2020). This list is, of course, far from complete, but it can illustrate why cooperation in large interdisciplinary networks *increases* complexity in correlation with the envisaged degree of precision and why the intuitive assumption to the opposite, namely that more precision would resolve misalignment and uncertainty, is ill-founded.

This leads to a final point to be made here. Epistemological uncertainties relate quite directly to the dynamic nature of PM as research field and hence resonate with its technoscientific potential. In principle, one would expect the scientific community to consolidate and stabilize relevant epistemological issues even though science, translational practice and technology constantly co-evolve dynamically. However, with regard to epistemological uncertainty, PM appears to represent a case of *disruptive innovation* (Aliferis & Adam, 2020). With this concept, we want do describe a development in which the high dynamics of technological innovation exceeds the possibility of epistemic stabilization thereby producing more uncertainty: In PM, due to its innovative nature and the exponential growth of data available for analysis thanks to more powerful technologies, ever new forms of uncertainty seem to arise from the evolving options and their combination. This is happening at a pace greater than can be contained through (ever outdated) evidence frameworks and standardization efforts, thereby constantly postponing the proleptic promise of epistemic certainty.

### Ontological uncertainty

**Fig. 1:**
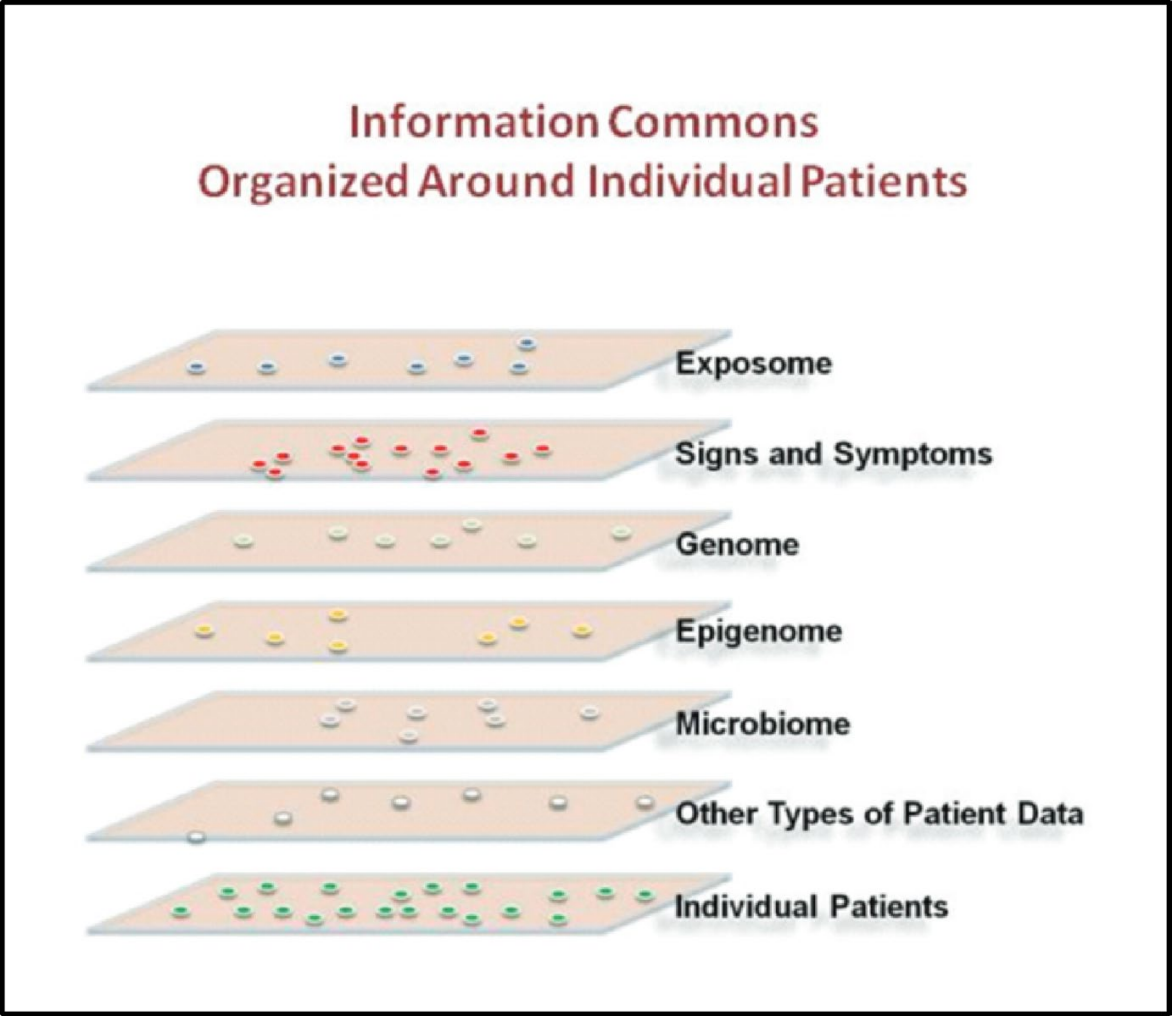
Levels of complexity in precision medicine (*Source* National Research Council Committee US, 2011, p. 17)

If EBM can be described as movement that bracketed ontological uncertainty by focusing on disease categories and statistical therapeutic efficacy, PM was announced as a re-opening of etiological and ontological questions, made possible by the advances of genetic research and genomic medicine (McCoy, 2020). However, this does not mean that there are no ontological sources of uncertainty in PM. To the contrary, PM raises substantial uncertainties in ontological respects, i.e. regarding the characteristics, composition and interactions of biomedically relevant entities and processes. One of the most relevant ontological challenges stems from the real-world complexity that PM attempts to capture. As indicated above, PM aims to take many more individual factors into account in research and treatment of diseases than traditional approaches (Kauffman et al., 2014, p. 29). These individual factors include not only information from medical records, but also about individual genomes, its derivates (epigenome, transcriptome, metabolome etc.) and information about lifestyle choices, dietary requirements and fitness habits. Ultimately, PM aims to be even more holistic by including exposome data (Fayet el al., 2024) about environmental factors, such as living conditions, air pollution and socio-economic status, as becomes clear from the influential vision of precision medicine developed by the *National Research Council Committee on a Framework for Developing a New Taxonomy of Disease* (2011) in the United States (see Fig. 1).

The attempt to capture more complexity regarding individual patient groups leads to uncertainty in two ways. *Firstly*, many of the PM levels described, even when viewed in isolation, are so complex that it is hardly possible to arrive at an understanding of the many interactions on these levels. For instance, the focus on the genome has led to an increase in uncertainty in diagnosis, as more and more (potentially) relevant genomic factors and interactions have been uncovered, so many that it seems unfeasible to handle them (Ji et al., 2018; Kerr et al., 2019). The molecular medicine that had started with the Human Genome Project and its promise to reveal decisive insights into disease trajectories has been converted into the systems biology of “intricate, redundant, and multidimensional” metabolic networks where mutations or knock outs only rarely lead to the expected observable consequences (Cooper & Paneth, 2020). *Secondly,* the interplay between different levels of complexity introduces further uncertainty; living organisms simply appear as being “too complex to yield up a set of fundamental laws” (ibid.). This interplay cannot be understood as simply additive. Rather, it follows an *emergentist* logic, making it extremely challenging if not impossible to deduce macro-level effects from the interaction of elements across different sub-levels. In consequence, the vast number of interactions may give rise to ambiguous—and unreliable—predictions of disease patterns (cf. Horesh Bergquist & Lobelo, 2018; Seymour et al., 2017).

With regard to the spectrum of uncertainties discussed here, ontological uncertainty seems to arise directly from the complexity of organisms and their environments and hence becomes a problem *because* PM attempts to capture it as holistically as possible. It may or may not be transient as only further research may show—in any case, ontological uncertainty results from innovative and rapidly evolving research rather than from merely insufficient efforts (we explore this further in the next section).

**Fig. 2:**
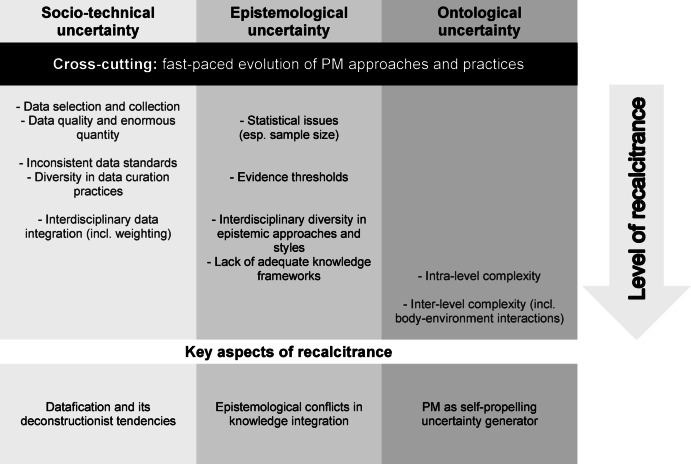
Sources of uncertainty in PM

Figure 2 is an attempt to sort the sources of uncertainty presented here according to their “level of recalcitrance”. This representation is necessarily imprecise and simplistic, but it can provide a rough orientation. However, it must be read against the background that there are a multitude of interrelations between these sources, which influence and shape each other in various ways. The mentioned “key aspects of recalcitrance” are elucidated in Sect. 3.3.

## Uncertainty as signature of precision medicine in practice

After identifying different sources of uncertainty on all three levels, the socio-technical, the epistemological and the ontological, we now zoom in at their interaction in translational research and clinical practice, using the example of molecular tumor boards in precision oncology (Sect. 3.1). Next, we zoom out again to discuss disciplinary transformations of the medical field (Sect. 3.2) and derive some general observations from our discussion of uncertainty that have implications for understanding the prospects and limitations of PM (Sect. 3.3).

### Zooming in: molecular tumor boards in precision oncology

(Precision) oncology is the field where PM has, at least partially, become clinical reality. In oncology, genetic profiling and other biomarkers have been introduced, mostly in wealthy countries, in clinical practice in form of molecular tumor boards (MTB) where clinical specialists meet with scientific experts from genetics, bioinformatics and systems biology for discussing treatment options for individual cases (Tsimberidou et al., 2023). As its name suggests, the MTB is the hallmark of the molecular approach of precision oncology, replacing clinical categories by genetic information and biomarkers (Luchini et al., 2020). This use of genomic medicine in oncology can serve as entry point for exploring the interplay of the different types of uncertainty in practice: Oncology was established, like most clinical disciplines and specialties, based on an organ-specific pathology. Cancer is the paradigmatic condition of Virchow’s cell- and organ-based pathology which starts with locating the organ of origin and the cell type of the tumor. The molecular profiling of tumors, however, has revealed genetic similarities between tumors from different body regions, heterogeneous organs and clinically vastly distant specialties. Due to these discoveries, clinical trials have meanwhile been shifted from being tumor-type-centered to “gene-directed, histology-agnostic” study designs (Tsimberidou et al., 2020), and a recent comment in *Nature* explains “why tumour naming needs to change” (André et al., 2024) in light of new developments in cancer research. A closer analysis of the operations of MTBs, however, has revealed that their implementation does not simply signal the advent of molecular and genomic medicine in clinical practice, but leads to complex hybridizations of basic and translational research with clinical practice with far-reaching epistemic and socio-technical effects: MTBs do not simply replace traditional disease definitions by more refined or pathophysiologically better justified, gene-based disease entities, they instead align genomic data with pharmacological efficacy profiles. “Actionability” as defined by available therapeutic drugs trumps disease classification, conflating diagnosis and therapy (Cambrosio et al., 2021).

New trials based on genetic markers may yield greater coherence with regard to actionability, but this challenges the traditional concept of disease as natural entity (and not merely their classification): A disease does no longer remain a fixed, biological, respective clinical entity, but turns into a moving target that arises from the available clinical data, biomarkers *and* economically feasible therapeutic opportunities (Gamma, 2017). The very ontology of cancer turns, at least partially, into an effect of the prevailing epistemological approaches (Pietarinen & Stanley, 2022).

Building on this *ontological* source of uncertainty, *epistemological* and *socio-technical* factors also play a role. This becomes clear in an ethnographic study of precision oncology by Even Chorev (2020) that describes how treatment recommendations are “paradoxically both immutable and uncertain” (p. 32). More specifically, this study reveals (similar to Bourret & Cambrosio, 2019 and Dam et al., 2022) how precision oncology practiced in MTBs despite—or rather because of—its high degree of individualization results in uncertainty regarding treatment decisions because of the intertwinedness of socio-technical and epistemological sources of uncertainty: Constantly evolving tumor profiling methods are generating ever more sub-types of oncological biomarkers with *potential* actionability. However, the relevance of a given biomarker or its weighting in light of contrasting biomarker information is often unclear. It can also shift during the therapeutic process and in light of ad hoc deliberations between molecular life scientists, bioinformaticians and clinicians on the relevance of specific genetic mutations and downstream pathways given experience with available cancer drugs and the clinical trajectory of the patient (cf. Bourret & Cambrosio, 2019). A key *epistemological* reason for this is that there is no evidence framework (in terms of trial rules) in place that is at the same time stable enough to provide sufficient guidance and flexible enough to deal with evolving tumor profiling practices and related ambiguities. As the cited empirical studies clearly show, “adaptable stability” has rather to be constructed in deliberative processes of the MTB.

Thus, at least in precision oncology, the individualization of disease which PM had envisioned and advertised as both, a clinical breakthrough by epistemic advances and a relief to patients, turns out to be a case for demonstrating how deeply ontological questions have got entangled with epistemological perspectives and the socio-technical conundrums of clinical practice – with the effect that uncertainty *increases* together with the new options available regardless of generally better outcomes in precision oncology (Larson et al., 2021). As such, this mini case study illustrates how the intricate interplay of the three sources of uncertainty leads to its emergence and persistence in clinical practice. A discussion whether this applies also to other branches of PM, where MTBs have meanwhile become a blueprint for practice (Distler et al., 2021; Schreiber et al., 2022), is beyond the scope of this paper (Cooper & Paneth, 2020; Khoury & Galea, 2016). The new insights into the complexity of pathological states and the interconnectedness of biological processes on the molecular level, however, turn pathophysiology into systems biology (O’Malley & Soyer, 2012; Walker et al., 2019) and replace the traditional “cascade-model” of disease by its conceptualization as complex “entanglement “ of body and environment (Boenink, 2017, p. 83f; Canali & Leonelli, 2022).

### Disciplinary transformations

PM begun as reinforcement of a mechanistic understanding of bodily processes, but the available research technologies have turned it into an ontologically flattened and open search for “patterns” and “correlations” as the new key terms in the epistemology of PM (Leonelli, 2016, chapt. 6). This leads to new uncertainties, namely whether and how significant correlations match with clinically relevant conditions: Patterns and correlations surface thanks to new techniques of data analysis and data visualization, their significance can be coupled to actionability; whether or how these patterns relate to states and stages of disease is no longer a morphological or materially tangible question as they merely capture relations of data and biomarkers (Baumgartner, 2021; Meunier, 2022). With the epistemological shift from deterministic explanations to dynamic patterns and correlations, PM has approached new insights, but apparently also tapped a rich source of uncertainty: Stephen Jay Gould once quipped that “variation itself is nature’s only irreducible essence” (Gould, 1991, p. 476). While currently ‘variation’ must be highlighted primarily as a challenging trend in the epistemology of PM research—recent advances in epigenetic, molecular and monitoring technologies ushering in new forms of experimentation, referred to as “data-driven science” (Strasser, 2012), “data harvesting” (Borck, 2022), “convenience experimentation” (Krohs, 2012) or “data-mining” (Boem & Ratti, 2017), yielding massive amounts of data rather than testing well-defined hypothesis (Meunier, 2022)—ultimately, this development may raise ontological questions about the role of variation in disease.

In addition to ontological and epistemological uncertainty, also socio-technical uncertainty does not only relate to the mundane or pedestrian questions of feasibility, practicality and expediency, but touches on more profound aspects. Of course, not every form of uncertainty in PM will be irresolvable. Some issues can be resolved or significantly reduced with improved data infrastructures, computational capabilities and better integrated meta-data; some uncertainties will simply become apparent as errors and gaps in biomedical knowledge when specific PM applications cannot deliver what they promise. And some of the epistemological black boxes will become more transparent with a better understanding of the underlying biological complexity, especially as “outliers” have been identified as potentially superior targets for PM interventions in oncology (Mateo et al., 2022). The development and implementation of innovative study designs is a promising sign (Ravi & Kesari, 2022); whether these will deliver, remains an open question.

Further, it is possible that future advances in AI technology and pattern recognition will ultimately be able to make sense of much more, although not all (Green & Vogt, 2016), of the ontological intra- and inter-level complexity we discussed above. *However*, even if we *can* make progress in pattern recognition and prediction using AI, this may ultimately come at the cost of a loss of transparency and hence: understanding. After all, better predictive capabilities do not necessarily involve “opening black boxes”. On the contrary, recent discussions about the limitations of *explainable AI* have made it clear that it will not always be possible to achieve greater transparency in successful AI systems (Meunier & Herzog, 2023). As a consequence, one form of uncertainty may be traded off for another type of uncertainty in this scenario.^2^

### Recalcitrant uncertainty in PM

As we tried to show, some forms of uncertainty will not be easy—or even impossible—to reduce as they are intrinsically linked to the PM research program. In our view, three aspects that we already touched upon play an important role in this respect. It will be useful to expand on these *key aspects of recalcitrance* (see Fig. 2) to fully understand their implications for the nature of uncertainty in PM: (1) PM has deconstructivist tendencies, (2) it is highly interdisciplinary, and (3) it is a self-propelling uncertainty generator.

1. Big data and data-intensive research in PM replace biological materiality to a degree, by breaking down disease into patterns, numbers and correlations. This is a driver of uncertainty, as this process seems to move from scientific reduction to *deconstruction*. This type of “datafication” (Beaulieu & Leonelli, 2022; Ruckenstein & Schüll, 2017) goes beyond mere (biomolecular) reduction, as it leads to a form of detachment from biological materiality and, to the extent this is already captured in PM, social reality. Operations are now primarily done on big assemblages of biological, medical and, at times, sociological data, which are in a sense granted their own kind of reality. However, the question arises to which degree re-integration from these operations can be realized, as we seem to be faced with a situation of dealing with an unmanageable number of puzzle pieces that cannot be put together anymore to form a meaningful picture. In other words, how can we maintain a theoretical framework that ties the things together if the puzzle pieces provide so many possible connections that we cannot form a coherent whole? This relates to the fundamental tension between the “generalization and individualization of inference” in PM (Gamma, 2017, p. 402): Does the belief in causal pathophysiology expire and does the intuition that a single patient must present as a coherent case fail? Perhaps the unlikely comparison with psychiatry is instructive here: Psychiatry abandoned a disease-based classification system and replaced it by multiple axes and scoring sets with the introduction of the DSM-III, hoping to arrive eventually at robust biomarkers (Hyman, 2021). After decades of futile research, however, the field seems to have meanwhile arrived at a broad consensus that there are no straight connections between molecular and neuroimaging biomarkers and many of the most relevant psychiatric conditions (Frisch, 2021; Kingdon, 2020). Could it be the case that the development of PM is being anticipated here?
2. Interdisciplinary cooperation has been heralded as motor of research productivity, allegedly arriving at deeper, more generalizable and more stable forms of knowledge (cf. Barry et al., 2008). PM started with that exact promise, and it was expected to arrive at a deeper understanding of disease processes. PM’s very productivity, however, points in the opposite direction: We highlighted knowledge integration as a key source of epistemological uncertainty. A central reason for the difficulty of integrating different types of evidence lies in the *interdisciplinary nature of PM*. It is challenging to synthesize conclusive information from the different types of evidence in the biomedical laboratory, in the clinic, from randomized trials and epidemiological studies “in the real world” etc. because these research areas work according to different research frameworks and established practices (Landecker & Panofsky, 2013). Research and scientific practice are aligned with different languages, theoretical assumptions and methodological preferences at all different sites; and there are also deeper and more implicit differences, including styles of reasoning and diverging standards of justification and assessment for evidential claims (cf. Oreskes, 1999, 2008). This does not mean that interdisciplinary knowledge integration is impossible, but it requires sophisticated socio-epistemic strategies (Bschir & Lohse, 2024)—more specifically, it requires strategies that go beyond improving data protocols by purification and statistical analysis by better tools. This applies all the more as the exponential growth of experimental and analytical possibilities outpaces rigid options for standardization.
3. PM can be described as a self-propelling uncertainty generator. The reason for this is that it does not only accelerate the usual scientific development logic, but changes it so that newly discovered findings reveal significant gaps in our knowledge about the ontological foundations of disease—which necessitates the ongoing and fundamental revision of medical knowledge. A telling example is the questioning of traditional organ-based disease classification by newly discovered pathophysiological pathways, linking hitherto remotely distant diseases in unexpected ways and offering promising new drug discoveries. However, this does apparently not lead to a new and stable disease classification, but to a more general and fundamental questioning of the conceptualization of diseases as stable entities (Dupré & Bertolaso, 2018). The observation that diseases develop and change over time (often in well-characterized ways, sometimes unexpectedly) is obviously not new, but if process and change dominate the picture it becomes questionable to conceive of disease as natural kinds (Lemoine, 2014). The significance of this has already been recognized for precision oncology (Militello & Bertolaso, 2022).

## Concluding thoughts: navigating uncertainty

In light of our analysis of the three sources of uncertainty and the reasons why at least some of them might turn out intransient, it seems justified to state that PM is characterized by profound uncertainties. It is less clear, however, what this means for an adequate understanding of PM. A natural conclusion that could be drawn from our analysis is that the medical approach we discussed in this paper should not be referred to as “precision medicine” at all, as this creates an image of the capabilities of PM that is misleading, at least for non-experts, such as patients and their families. Further, if we cannot avoid it, how can we successfully navigate uncertainty in PM? What implications does our analysis have for translational research and clinical practice? While we cannot offer fully satisfactory answers to these questions in this paper, we propose a few possible ways forward.

One response to the problem of uncertainty in PM could be to create substantially simplified models instead of “supermodels”, i.e. models that represent only a limited subset of layers and elements in order to avoid dealing with too much complexity delivered by omics systems and real world data regimes. Perhaps it will be effective to focus on only *some* biomarkers, on already existing clinical data and on robust self-reported outcomes. This strategy, however, carries the risk of excessive reduction, perhaps even a regression from PM to genomic medicine (Friedman, 2022; Tabery, 2024). There is always the possibility that it is the most crucial layers and elements that will be omitted, e.g. when qualitative knowledge about life circumstances is excluded from the analysis because it does not neatly fit into what works from the perspective of a simplified data model.

If the path of simplification is not to be taken, significantly more resources will have to be channeled into the development of *resilient* evidence frameworks and synthesis strategies. The key point to be stressed here is that such strategies should not only be conceptualized at the level of data standards, technological solutions and mere protocols. Rather, strategies to evidence integration will need to (a) consider challenges stemming from heterogeneous and instable ontologies, (b) manage different epistemological affinities in interdisciplinary settings (learning from the field of interdisciplinarity studies), and (c) foster a new kind of epistemic humility in PM that acknowledges *limitations of pluralistic knowledge integration* and finds better ways to make different forms of uncertainty visible and explicit.

As with EBM, we will not see a “pure” PM-clinic. There are always elements of practical routines, tacit knowledge and clinical judgement that cannot (and must not) be abandoned, but will need to be amalgamated with PM strategies. In our view, this is clearly positive because it means that strategies already in use for dealing with uncertainty in the clinic—seeking multidisciplinary feedback, acknowledging epistemic limitations in view of “wicked” health problems etc. (Scott et al., 2023; Hoeyer, 2023, chapt. 5)—can be utilized. On the other hand, it will also be necessary to devise and to experiment with new practical ways of managing uncertainty in the context of clinical decision-making. It remains to be seen, for example, whether opaque AI analyses will represent insurmountable obstacles here or to what extent the trust relationship between doctor and patient will have to be reshaped and re-consolidated in order to accommodate the inclusion of black box evidence. The fact that this evidence might increasingly be delivered not by scientific institutions, but by powerful IT companies pushing into the health sector, requires further consideration and debate to a degree that we can only mention this aspect here.

Finally, navigating uncertainty in PM effectively could mean to learn from best practice approaches in MTBs and their analogues in other branches of PM for developing an open uncertainty communication as integrated element in shared decision-making where “the clinical encounter provides an occasion for the doctor to go over all the various evidence, sort it *in a meaningful manner* and articulate explicit reasons for *including or excluding certain findings or options*—even if no coherent explanation is found” (Scott-Fordsmand & Tybjerg, 2023, p. 17, our emphasis). Perhaps Osler was a visionary and PM is the “science of uncertainty,” which is why clinical decision-making needs more than rigid guidelines, namely an art of probability—and of ambiguity, we might add.

## Data Availability

Not applicable.

## Footnotes

1 “Limited success” as there remain a multitude of challenges for precision oncology, see, e.g., Plutynski (2022) and our discussion below.

2 There are other cases in which the reduction of uncertainty will go hand in hand with epistemic trade-offs. Consider the issue of statistical uncertainty which results from the extreme individualization of PM. Clearly, it will be possible to counter this problem by enlarging the respective sample sizes in clinical trials. The same applies to filtering out spurious biomarkers that only *appeared* to be actionable in exploratory treatment attempts of small patient groups. But this move would contradict the logic of PM, as it presupposes limits to individualization and can no longer set perfect personalization as the ultimate goal.
